# Is Development of a Vaccine against *Cryptococcus neoformans* Feasible?

**DOI:** 10.1371/journal.ppat.1004843

**Published:** 2015-06-18

**Authors:** Chrissy M. Leopold Wager, Floyd L. Wormley

**Affiliations:** 1 Department of Biology, The University of Texas at San Antonio, San Antonio, Texas, United States of America; 2 The South Texas Center for Emerging Infectious Diseases, The University of Texas at San Antonio, San Antonio, Texas, United States of America; Duke University Medical Center, UNITED STATES

## Introduction

*Cryptococcus neoformans*, the predominant etiological agent of cryptococcosis, is an encapsulated fungal pathogen that can cause fungal pneumonia and life-threatening infections of the central nervous system (CNS) [1]. *C*. *neoformans* can be found ubiquitously throughout the environment [1]. Inhalation of airborne yeast or desiccated basidiospores typically results in asymptomatic disease or dormant infections; however, progression towards clinical disease commonly occurs in persons with severely compromised immune responses. Global estimates suggest that 1 million cases of cryptococcal meningitis occur each year, resulting in approximately 625,000 deaths [2]. Morbidity and mortality rates due to cryptococcosis are significantly higher in resource-limited settings and in individuals with impaired CD4^+^ T cell-mediated immune responses (reviewed in [3–5]). Current therapies are often rendered ineffective because of the development of drug resistance by *C*. *neoformans*, drug toxicity, and treatment cost. Thus, a need remains for a cost-effective approach to prevent cryptococcosis.

## Is Developing a Vaccine against *C*. *neoformans* a Wise Thing to Do?

Simply restoring immune function in immune-compromised individuals has resulted in a decline in cryptococcosis; however, *Cryptococcus*-related immune reconstitution inflammatory syndrome (IRIS), which is also life threatening, is observed in a significant percentage of HIV^+^ individuals receiving highly active antiretroviral therapy (HAART) and in solid organ transplant recipients following administration of antifungal drugs and a reduction in immune-suppressive therapy [6]. The need for prophylactic measures to prevent cryptococcosis among immune-compromised persons is clearly evident. However, considering that the very arm of the immune response tasked with defending against *C*. *neoformans* is absent in the majority of individuals at the highest risk for developing cryptococcosis leads to questions regarding the feasibility of developing a vaccine that is effective in immunocompromised patients. Also, will the protective immune response generated by an anticryptococcal vaccine have the unintended consequence of predisposing the vaccinated population to *Cryptococcus*-related IRIS, thus augmenting host damage?

## Can Protection Be Achieved in Immune-Compromised Patients?

Clinical and experimental evidence suggests that protective immunity against cryptococcosis is dependent upon Th1-type CD4^+^ T cell-mediated immune responses (reviewed in [5]). Th1-type CD4^+^ T cells orchestrate protective immune responses against *C*. *neoformans* through the generation of a Th1-type cytokine profile characterized by the production of interleukin (IL)-2, IL-12, tumor necrosis factor (TNF)-α, and interferon (IFN)-γ, which induce lymphocyte and phagocyte recruitment to the site of infection and increase phagocyte uptake and killing of *C*. *neoformans*. Consequently, it may seem counterintuitive to suggest that development of an effective anticryptococcal vaccine that (1) confers protection in persons with low CD4^+^ T cell counts (i.e., HIV^+^ patients) and (2) induces protection that endures during the subsequent development of immune suppression is feasible.

Antibody-mediated immunity (AMI) has long been considered an obvious target mechanism for inducing vaccine-mediated protection against cryptococcosis in patients with suppressed cell-mediated immunity. HIV is associated with B cell defects that have been linked to increased susceptibility to cryptococcosis [7]. Studies focusing on AMI to cryptococcosis have provided promising results. Monoclonal antibodies (mAb) against the *C*. *neoformans* capsular polysaccharide glucuronoxylomannan (GXM) are capable of reducing organ cryptococcal burden and prolonging survival in mice [8]. Furthermore, protective antibodies against *C*. *neoformans* are able to aid in phagocytosis, modulate the inflammatory response, and alter gene expression of the yeast, rendering it more susceptible to antifungal drugs [7]. This demonstrates potential for antibodies as effective treatment options against cryptococcosis; however, more study is needed to evaluate the efficacy of antibodies to control cryptococcosis in immunocompromised hosts.

Previous studies by Huffnagle et al. have shown that CD8^+^ T cells may compensate for the loss of CD4^+^ T cells to facilitate protection against cryptococcosis in an IFN-γ-dependent manner [9]. Similarly, immunization with a *C*. *neoformans* strain genetically engineered to produce IFN-γ, designated H99γ [10], can induce protective immunity against cryptococcosis in mice depleted of CD4^+^ T cells [11]. Immune-competent mice immunized with *C*. *neoformans* H99γ and subsequently rendered both CD4^+^ and CD8^+^ T cell deficient (>98% depletion of each population) prior to and during challenge with wild-type (WT) *C*. *neoformans* were completely protected, as evidenced by 100% survival and sterilizing immunity [10,11]. Protective immunity has been observed up to 100 days postimmunization with the IFN-γ-producing strain [12]. These studies provided proof of concept that vaccines designed to combat *C*. *neoformans* infections are capable of inducing potent, long-lasting anticryptococcal immunity in immune-compromised patients.

## Is the Phagocyte’s (Macrophage or Dendritic Cell) Activation Status Critical for Protection against *C*. *neoformans*?

**Fig 1:**
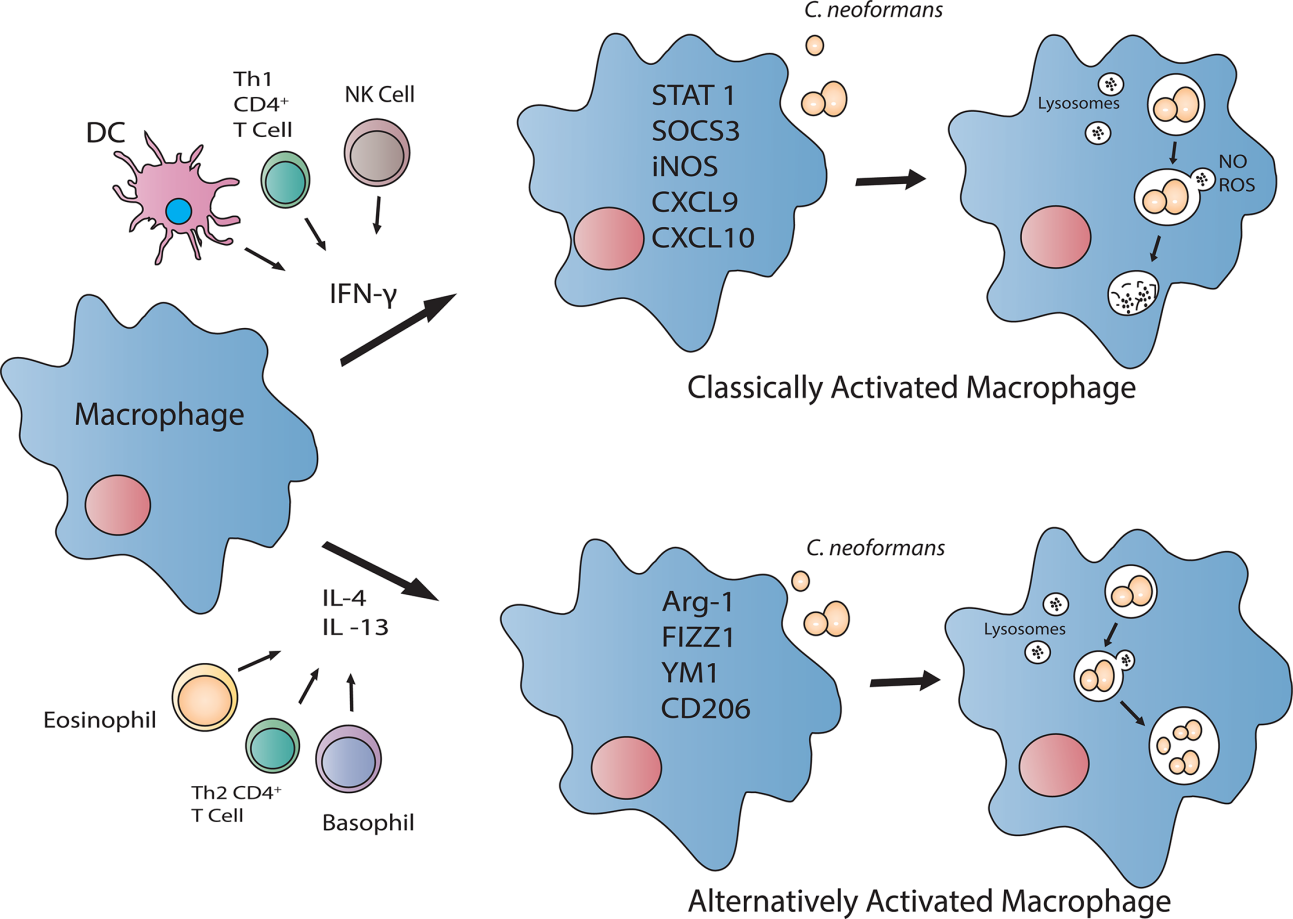
The activation status of the macrophage directly influences cryptococcal killing. In the presence of Th1-type cytokine IFN-γ, macrophages polarize to a classically activated (M1) phenotype. These macrophages produce reactive oxygen species (ROS) and NO, which contribute to their anticryptococcal activity. However, when the Th2-type cytokines IL-4 and/or IL-13 are more prevalent, macrophages polarize toward an alternatively activated (M2) phenotype. M2 macrophages do not have anticryptococcal activity and are permissive to intracellular proliferation of *C*. *neoformans*.

Since inhalation is the principle route for entry of *C*. *neoformans* propagules, resident pulmonary macrophages and dendritic cells (DCs) are well situated to contain the pathogen and prevent dissemination. Macrophages, and perhaps DCs, are capable of polarizing toward a fungicidal, classically (M1) activated phenotype or a cryptococcal growth-permissive, alternatively (M2) activated phenotype, depending on the cytokine milieu (Fig 1) (reviewed in [5,13]). Pulmonary infection with *C*. *neoformans* in mice typically induces Th2-type cytokine responses and M2 macrophage activation, resulting in uncontrolled fungal growth, dissemination, and disease exacerbation [14,15]. In stark contrast, pulmonary inoculation with *C*. *neoformans* H99γ results in Th1-type and IL-17A cytokine responses, M1 macrophage activation, and resolution of disease [16,17]. Additionally, mice protectively immunized with *C*. *neoformans* strain H99γ and subsequently challenged with WT *C*. *neoformans* develop an M1 macrophage activation phenotype with enhanced fungistasis and nitric oxide (NO) production associated with enhanced signal transducer and activator of transcription 1 (STAT1) signaling [18].

During inoculation with *C*. *neoformans* strain H99γ, STAT1^-/-^ mice show a decrease in M1 and increase in M2 macrophage activation markers and uncontrolled intramacrophage proliferation of the yeast, correlating with increased pulmonary and central nervous system (CNS) fungal burden and 90% mortality [19]. Furthermore, the increased intramacrophage cryptococcal growth in STAT1^-/-^ mice coincided with decreased NO production [19]. These studies indicate that NO production by M1 macrophages is critical for their anticryptococcal activity.

DCs phagocytose and kill *C*. *neoformans* by oxidative and nonoxidative methods and subsequently present antigen to T cells to help guide the adaptive response [20–23] via the production of cytokines and chemokines that induce cellular infiltration and the production of antimicrobial peptides and inflammatory mediators (reviewed in [24,25]). Recognition of cryptococcal CpG DNA by toll-like receptor (TLR) 9, for example, results in the production of IL-12p40 and co-stimulatory molecule CD40 in murine DCs [26]. Similar to the activation phenotype observed with macrophages, DCs exhibit characteristics akin to an inflammatory (DC1) or alternative (DC2) activation phenotype [27,28]. Specifically, stimulation of DCs with IL-4 induces expression of multiple alternative activation markers both in vivo and in vitro, albeit with a different expression pattern compared to that of macrophages [28]. Concurrent stimulation of DCs with IL-4 and TLR ligands including lipopolysaccharide (LPS) and CpG DNA results in boosted IL-12p70 and inhibited IL-10, resistin-like molecule α (RELMα), and chitinase3-like 3 (Chil3 or YM1) production [28]. The ability of DCs to respond to antigen by producing cytokines and chemokines that drive protective immune responses makes the targeting of these innate cells an attractive option for antifungal vaccine design. We postulate that the efficacy for a vaccine to enhance anticryptococcal activity of innate cell populations, particularly when T cell numbers are diminished, will depend upon the cytokines/chemokines produced by DCs and their subsequent influence on effector cell responses.

## Could Innate Cells “Trained” to Provide Protection against Cryptococcosis Be a Potential Vaccine Strategy?

Studies by Netea et al. showed that an initial exposure of monocytes/macrophages to a microbe or antigen enhances their innate immune responses against restimulation, a concept termed “trained immunity” [29,30]. Altogether, the studies indicate that monocytes and macrophages exhibit memory-like responses following exposure to the fungal cell wall component β-glucan and the enhanced responses are associated with changes in the epigenetic programming of the monocytes [31]. These findings suggest that therapies targeting monocytes and/or macrophages could potentially provide protective immunity, even in immune-compromised patients; however, the duration of these “trained” effects beyond 4–8 weeks remains to be assessed. Immune-based therapies and/or vaccines designed to augment macrophage responses against *C*. *neoformans* are plausible strategies considering the critical role of STAT1-mediated M1 macrophage activation in mediating protection. Recent studies demonstrate that macrophages polarized with IL-4 to an M2 phenotype can be repolarized to a functional M1 phenotype upon exposure to IFN-γ [32], demonstrating the plasticity of macrophage activation and their susceptibility to therapeutic manipulation. In theory, DCs “trained” to polarize towards an inflammatory phenotype are prime candidates to sustain a Th1-type cytokine environment that is critical for maintenance of an M1 macrophage activation phenotype in T cell-deficient hosts. However, will the induction of vaccine-mediated immune responses in immune-deficient hosts predispose these individuals to *Cryptococcus*-related IRIS? This may be best tested in an experimental model system by looking for signs of IRIS following immunization of a T cell-depleted host and subsequently allowing the host to reconstitute their T cell populations during the recall response to *C*. *neoformans*. In human patients, sensitive tests designed to detect cryptococcal antigen could be used to screen asymptomatic individuals in order to reduce the risk of potentially provoking cryptococcal-induced IRIS following reconstitution of the immune system. The absence of IRIS will support the efficacy and safety of vaccine strategies to mediate protection in immune-compromised hosts. In contrast, any observation of IRIS may provide a model system by which to test therapies designed to ameliorate complications due to IRIS in patients.

## Is a Vaccine against Cryptococcosis Feasible?

The discussion and evidence provided above suggest that a vaccine is possible; however, specific considerations must be made when designing immunotherapeutics. Patients lacking intact T cell responses will undoubtedly suffer from reduced memory T cell responses, rendering common vaccine strategies ineffectual. Thus, novel therapies that target innate immune cells such as macrophages and DCs have the potential to mediate protective immunity against *C*. *neoformans*. Recent studies indicate that these phagocytes are capable of trained immunity exhibiting enhanced protective responses upon secondary exposure. However, it is not yet known how long-lasting these “trained” effects are and if this approach to vaccine design is plausible. Nonetheless, protective immunity has been observed in the absence of traditional adaptive immunity, thus providing proof of concept that vaccines targeting innate immune responses may prove effectual in prevention or treatment of cryptococcosis.
